# White matter trajectories in Down syndrome and Alzheimer's disease: Insights from diffusion tensor–based morphometry

**DOI:** 10.1002/alz.70382

**Published:** 2025-06-12

**Authors:** Fedal Saini, Phoebe Ivain, Mina Idris, Jasmine Wells, Leda Bianchi, Miren Tamayo‐Elizalde, Flavio Dell'Acqua, André Strydom

**Affiliations:** ^1^ Institute of Psychiatry Psychology & Neuroscience Department of Forensic and Neurodevelopmental Sciences King's College London London UK; ^2^ Institute of Psychiatry Psychology & Neuroscience, Natbrainlab King's College London London UK; ^3^ Oxford Institute of Clinical Psychology Training and Research Isis Education Centre Warneford Hospital Oxford UK; ^4^ Institute of Psychiatry Psychology and Neuroscience Sackler Institute for Translational Neurodevelopment King's College London London UK

**Keywords:** Alzheimer's disease, dementia, diffusion magnetic resonance imaging, diffusion tensor imaging, diffusion tensor–based morphometry, Down syndrome, neurodegeneration, structural connectivity, trisomy 21, white matter

## Abstract

**INTRODUCTION:**

Diffusion magnetic resonance imaging studies investigating Down syndrome (DS) and Alzheimer's disease (AD) have mainly relied on white matter (WM) skeleton‐based techniques, potentially overlooking broader WM architecture.

**METHOD:**

We applied diffusion tensor–based morphometry (D‐TBM), a novel whole‐volume WM registration technique, to characterize WM properties in DS. Between‐ and within‐group analyses were conducted in 51 adults with DS and 35 controls, divided into two age groups, using diffusion tensor imaging (DTI)–derived metrics and local volumetric changes.

**RESULTS:**

DS participants exhibited extensive volumetric and DTI‐based differences affecting association fibers and commissures. Within‐group comparisons revealed further changes in older DS participants in these fibers. Reduced axial diffusivity (AxD) in temporal and commissural WM was reported for the first time in DS.

**DISCUSSION:**

DTI changes in the older DS cohort affect WM structures supporting language, memory, and executive functions and may be due to AD‐related atrophy. Reduced AxD may reflect neuroinflammation or atypical WM development.

**Highlights:**

- Diffusion tensor–based morphometry (D‐TBM) was applied for the first time in Down syndrome (DS) adults.
- Diffusion tensor imaging alterations in DS affect structures for language, memory, and executive functions.
- Increased radial diffusivity and mean diffusivity in older DS adults highlight Alzheimer's disease (AD)‐related neurodegeneration in key tracts.
- AD in DS affects commissural structures, including the genu of the corpus callosum.
- Axial diffusivity reductions in DS may indicate neuroinflammation.

## 1 BACKGROUND

People with Down syndrome (DS) have an exceptionally high risk of developing Alzheimer's disease (AD), primarily due to the triplication of the amyloid precursor protein gene on chromosome 21.^1^ This triplication leads to overproduction of amyloid beta (Aβ) protein, resulting in AD neuropathology starting by the mid‐30s in most individuals with DS,^2^, ^3^ with dementia developing in the majority by age 60.^4^

Though AD is often seen as a gray matter (GM) disease, white matter (WM) damage also occurs early in its progression. Diffusion magnetic resonance imaging (dMRI) studies have shown alterations in several brain regions, including the frontotemporal lobes and the corpus callosum (CC) in sporadic AD (sAD); for a review, see Caso et al.^5^ These studies demonstrate the sensitivity of dMRI in detecting subtle WM changes not visible with traditional structural MRI, supporting its potential as a biomarker for AD.

Research using dMRI in DS, however, is still in its early stages. Studies to date have identified diffusion alterations in DS compared to typically developing (TD) controls, particularly in the CC and long‐range associative cortico–cortical connections; for a review, see Saini et al.^6^ Additionally, dMRI has been useful in distinguishing individuals with DS who have AD from those who do not, particularly through changes in the CC and temporal WM.^7^ Associations have also been found among diffusion measures, brain amyloid burden,^8^ and cognitive decline.^7^, ^9^, ^10^, ^11^

Most of these studies have relied on tract‐based spatial statistics (TBSS),^12^ a spatial normalization technique that, while well established, focuses only on the most anisotropic WM regions, potentially overlooking much of the brain's WM architectural complexity. Specifically, TBSS focuses on the WM skeleton, a one voxel wide structure derived by selecting the voxel with the highest fractional anisotropy (FA) value across the tract, thus capturing only the core of each major WM tract. Moreover, when registering data to a template space, TBSS relies solely on the tensor's scalar information (i.e., FA), which reflects the amount of diffusion. While effective, this registration does not account for the directional information (i.e., vectorial information) of WM across different subjects, and registration in regions of low anisotropy, such as GM or cerebrospinal fluid (CSF), may be suboptimal.^13^, ^14^

To address these limitations, in this study we have used diffusion tensor–based morphometry (D‐TBM). D‐TBM is a novel morphometric technique that integrates both scalar and vectorial information from the diffusion tensor (i.e., amount and direction of diffusion, respectively), providing greater anatomical accuracy when registering brain data to standard space. This ultimately enables increased sensitivity and specificity when performing statistical analysis.^15^, ^16^ Moreover, D‐TBM allows for voxel‐wise analysis across the entire WM volume, capturing data from the outermost areas of tracts. This is particularly important in studying neurodevelopmental and neurodegenerative conditions like DS and AD, in which WM changes may not be confined to the core of tracts. Last, a further advantage of D‐TBM is the incorporation of the Jacobian determinant (Ln‐J), which measures local deformation or volume changes by showing how the imaged tissue has expanded or contracted during registration.^17^, ^18^ This provides an additional layer of information, allowing for simultaneous assessment of both tissue microstructure properties and WM morphology.

In this study we aim to characterize the developmental and neurodegenerative trajectories of WM in individuals with DS using advanced dMRI registration techniques. We compared 51 adults with DS to 35 age‐ and sex‐matched TDs, focusing on DTI‐derived scalar metrics and local volumetric changes using D‐TBM. Participants were divided into two age groups: younger adults, to explore trisomy 21–related neurodevelopmental effects, and older adults, to investigate AD‐related neurodegeneration in addition to neurodevelopmental effects in DS. Additionally, within‐group analyses were conducted to examine age‐related WM differences in DS and TDs (older vs. younger). By combining DTI‐derived scalar metrics with D‐TBM's capacity to detect local volumetric changes, this study aimed to provide enhanced understanding of WM alterations in DS, encompassing both neurodevelopmental and neurodegenerative processes.

## 2 METHOD

### 2.1 Study design and ethical considerations

This cross‐sectional study compared dMRI data from adults with DS and TDs. MRI data for all participants with DS were collected under the LonDownS‐PREVENT study, with ethical approval from the North Wales West Research Ethics Committee (IRAS: 120344, study number: 13/WA/0194), and for controls from studies approved by the King's College London Health Faculties Research Ethics Subcommittee (HR/DP‐22/23‐35003) and the SAGA‐B project, approved by the King's College London Human Research Ethics Committee (HR‐17/18‐5720). All participants were scanned using identical protocols on the same machine.

Participants with DS were recruited from across England and Wales, particularly greater London and southeast England. Informed consent was obtained, in keeping with the provisions of the UK Mental Capacity Act 2005. For participants with DS with decision‐making capacity, written consent was provided; for those without capacity, a consultee familiar with their preferences signed on their behalf. Standard informed consent procedures were followed for TD participants.

### 2.2 Participants and procedure

All participants were aged ≥ 16 years and met standard MRI safety criteria. Individuals with acute physical or mental health conditions were excluded. Participants with DS had a confirmed clinical diagnosis of trisomy 21, validated through genetic testing via saliva or blood samples. Additionally, participants unable to adhere to experimental instructions were excluded.

A total of 75 participants with DS were initially recruited, but 20 were excluded prior to preprocessing. Of these, nine participants (seven females; ages 24–57 years) were unable to complete the MRI scan, including one participant (53 years old) with a dementia diagnosis. Another six participants (three females; ages 34–64 years) completed some MRI sequences but did not proceed to diffusion‐weighted imaging (DWI). A further five participants (four females; ages 20–65 years) successfully underwent diffusion acquisition; however, the data quality was insufficient for analysis due to excessive movement noise. After preprocessing, an additional four participants (two females; ages 24–51 years) were excluded due to excessive motion, resulting in a final sample of 51 adults with DS (mean age = 37.41 years, standard deviation [SD] = 9.77; 28 females). The TD group included 35 participants (mean age = 38.86 years, SD = 10.01; 16 females). Demographic and general medical information from all participants were collected.

### 2.3 MRI acquisition

DWI data were acquired following the internationally standardized “Diffusion ADNI‐3 Advanced” protocol. All imaging data were acquired using a research‐dedicated GE Discovery MR750 3T MRI scanner (General Electric) at the Centre for Neuroimaging Sciences, King's College London. DWI data were acquired using a cardiac‐gated, single‐shot spin echo EPI Multi‐Band, Multi‐shell acquisition protocol using a 32‐channel Nova head coil with the following parameters: echo time = 71 ms, repetition time = 3300 ms, 120 total diffusion‐weighted directions divided into 12 directions at *b* = 500 s/mm^2^, 48 directions at *b* = 1000 s/mm^2^, and 60 directions at *b* = 2000 s/mm^2^; 72 slices with a voxel size of 2 mm^3^ isotropic; field of view of 232 × 232 × 160 mm^3^; and an acquisition time of ≈ 7 minutes and 10 seconds. An additional 12 non‐diffusion weighted (b0) volumes were also acquired interleaved with the DWI acquisition, while 6 non‐diffusion‐weighted volumes were collected with reversed phase encoding EPI to allow for susceptibility correction of the data.

**RESEARCH IN CONTEXT**

1. **Systematic review**: The brain's white matter (WM) can be studied using diffusion magnetic resonance imaging (dMRI). Previous dMRI studies in Down syndrome (DS) and Alzheimer's disease (AD) have reported alterations in major connective structures. However, most studies relied on registration techniques that capture only the core aspects of WM, potentially overlooking broader and more subtle WM changes.
2. **Interpretation**: Using a novel dMRI registration technique that enables whole‐volume WM analysis with enhanced anatomical precision, we identified extensive diffusion and volumetric WM changes in DS, particularly in tracts associated with language, memory, and executive functions. Notably, we reported reduced axial diffusivity (AxD) in DS, potentially reflecting either atypical WM neurodevelopment or early neuroinflammatory processes, which may serve as a marker of WM differences in DS.
3. **Future directions**: Future studies of AD should explore more advanced diffusion modeling and the role of AxD in this population should be further investigated.

### 2.4 Diffusion MRI data pre‐processing and tensor fitting

The quality of the DWI data was assessed using a locally developed pipeline that followed current best practices recommended by the International Society for Magnetic Resonance in Medicine (ISMRM) and the Alzheimer's Disease Neuroimaging Initiative consortium. Initially, DWI data were visually inspected for major issues, such as data corruption and protocol inconsistencies. The data were then preprocessed through the following steps: de‐noising using the Marchenko–Pastur method;^19^ Gibbs ringing correction;^20^ estimation of susceptibility distortion fields using top‐up;^21^ and correction for motion, eddy currents, outlier detection and replacement,^22^ susceptibility distortions, and intra‐volume slice motion, performed with the eddy tool from the FSL software package.^23^ To ensure data quality, datasets with absolute head motion exceeding 2 mm were excluded from analysis.

After preprocessing, the diffusion tensor model was fitted to the diffusion data using non‐linear least squares (NLLSs) fitting implemented in the TORTOISE software.^24^ From the estimated tensor, several diffusion metrics were extracted, including FA, mean diffusivity (MD), radial diffusivity (RD), and axial diffusivity (AxD).^25^

### 2.5 Brain templates and D‐TBM spatial registration

Template generation and spatial registration, essential for D‐TBM analyses, were performed using the DR‐TAMAS (Diffusion Registration, Template, and Atlas‐based Morphometry Analysis System) tool, a diffeomorphic registration method for DTI data. This method uses all tensor features, instead of derived scalars such as anisotropy, ensuring a more accurate registration and reversible mapping between images. It preserves complex brain structures and topology, enabling high‐quality registration across WM, GM, and CSF regions.^26^

To account for atypical neurodevelopment in individuals with DS, a study‐specific brain template was generated to improve inter‐subject registration and minimize geometric distortions. Given the substantial morphological differences between DS and TD brains—including differences in overall brain shape, cortical folding, and ventricular enlargement^27^—registering one group to a template derived solely from the other can introduce significant non‐linear warping. This can lead to interpolation‐induced errors and affect voxel‐wise analyses of diffusion properties. To mitigate these issues, we created a symmetric, study‐specific template using diffusion‐weighted images from both DS and TD participants, providing a population‐representative anatomical reference that reduces excessive deformation during registration.[Table alz70382-tbl-0001]

The template was generated from a sub‐cohort of 20 DTI maps of younger individuals with DS (mean age = 26.7; SD = 5.71; age range = 17–34; *n* = 12 females; *n* = 10 mild intellectual disability [ID]; *n* = 8 moderate ID; *n* = 2 severe ID), who did not have clinical features of AD, and from a sub‐cohort of 15 younger TD participants (mean age = 29.33; SD = 4.86; age range = 19–37; *n* = 9 females). The study‐specific brain template created in this study is available from the corresponding author upon reasonable request.

### 2.6 Analyses

For the purpose of differentiating WM changes related to the triplication of chromosome 21 from those caused by AD neuropathology, participants were divided into two age brackets: those aged ≤ 35 years, and those aged ≥ 36 years. This cutoff was selected based on literature suggesting that typical AD neuropathology develops in the majority of individuals with DS after their mid‐30s.^2^, ^3^ Two sets of analyses were then conducted: between‐group analyses comparing younger adults with DS to younger TD adults, and older participants with DS to older TD adults, as well as within‐group analyses. The latter examined age‐related WM differences within each population by comparing younger and older adults with DS and younger and older TD adults separately.

Volumetric (i.e., Ln‐J) and FA changes were analyzed as the primary outcomes to assess structural and DTI‐based differences between participants with DS and TDs across the two age groups and in within‐group comparisons. If significant differences in FA were identified, post‐hoc analyses were conducted to explore further WM differences using MD, RD, and AxD metrics. For all between‐group analyses, age and sex were included as covariates, while for within‐group analyses, only sex was used as a covariate.

A WM mask was created by thresholding FA values of the brain template at 0.2. Voxel‐wise statistical analysis was performed using FSL's randomize,^28^ a permutation‐based inference method for non‐parametric statistical thresholding, combined with threshold‐free cluster enhancement (TFCE) and corrected for multiple comparisons using family‐wise error (FWE) at *P*FWE < 0.01.

## 3 RESULTS

### 3.1 Demographics and study sample

Demographic information for all participant groups is presented in Table 1. Initially, the study included 55 participants with DS and 35 TDs. After dMRI pre‐processing, four datasets from participants with DS were excluded due to movement artifacts, leaving a final sample of 51 adults with DS and 35 TDs. The excluded participants (*n* = 4) were two males aged 24 and 29 years and two females aged 31 and 51 years, all with moderate ID. None of these excluded participants had dementia. Participants were then divided into two age groups: ≤ 35 years, and ≥ 36 years.

**TABLE 1 alz70382-tbl-0001:** Participant demographics.

Age group	Younger adults (<36 years)		Older adults (≥36 years)	
Diagnostic group	DS	TD	DS	TD
Females	15	8	13	8
Males	10	5	13	14
Total	25	13	26	22
Mean age ± SD [min–max] (years)	26.88 ± 5.93 [19–33]	28.15 ± 4.03 [19–33]	45.73 ± 6.22 [36–58]	47.63 ± 7.83 [37–58]
ID level (mild, moderate, and severe)	12/8/2	N/A	12/14/3	N/A

*Note*: Summary of demographic characteristics for younger and older adults with DS and TD controls. Age is reported as mean ± SD with the full range in brackets. ID levels are provided for DS groups only.

Abbreviations: DS, Down syndrome; ID, intellectual disability; SD, standard deviation; TD, typically developing controls.

For the younger adult groups (i.e., ≤ 35 years), the DS group consisted of *n* = 25 participants (mean age = 26.88 years, SD = 5.93, age range = 19–33 years; *n *= 15 females), with 12 participants having mild ID, 8 moderate ID, and 2 severe ID. The younger TD group comprised *n =* 13 participants (mean age = 28.15 years, SD* =* 4.03, age range = 19–33 years; *n* = 8 females). None of the younger participants with DS or TD had dementia. Wilcoxon rank sum tests showed no significant age difference between the younger groups (*W =* 153, *p =* 0.7), and chi‐squared tests found no significant difference in sex distribution (*χ2 =* 0, *df =* 1, *p =* 1.0).

For the older adult groups (i.e., ≥ 36 years), the DS group included *n =* 26 participants (mean age = 45.73 years, SD = 6.22, age range = 36–58 years; *n* = 13 females), with 12 participants having mild ID, 14 moderate ID, and 3 severe ID. Additionally, four participants in this group were undergoing clinical evaluation for suspected dementia, though no formal diagnosis had been made at the time of participation. The TD group consisted of *n =* 22 participants (mean age = 47.63 years, SD* =* 7.83, age range = 37–58 years; *n* = 8 females). None of the TD older adults had dementia. Wilcoxon rank sum tests revealed no significant age difference (*W =* 245.5, *p =* 0.4), and chi‐squared tests found no significant difference in sex distribution (*χ2 =* 0.43157, *df =* 1, *p =* 0.5).

### 3.2 Between‐group comparisons results: younger adults

In the younger cohorts, whole‐brain analysis revealed extensive Ln‐J reductions in individuals with DS compared to TDs (*p* < 0.01), predominantly affecting frontotemporal and cerebellar areas. Significant FA reductions (*p < 0.01*) were also observed in the DS group, impacting all major long association and projection fibers.

Post hoc analyses found no significant differences in MD between the two groups. However, a significant increase in RD (*p *< 0.01) was identified in the DS group, primarily localized to the anterior limb of the internal capsule bilaterally, the right external capsule, fornix, and pons. In contrast, AxD was significantly reduced (*p *< 0.01) in the DS group, affecting temporal and deep WM regions.

**FIGURE 1:**
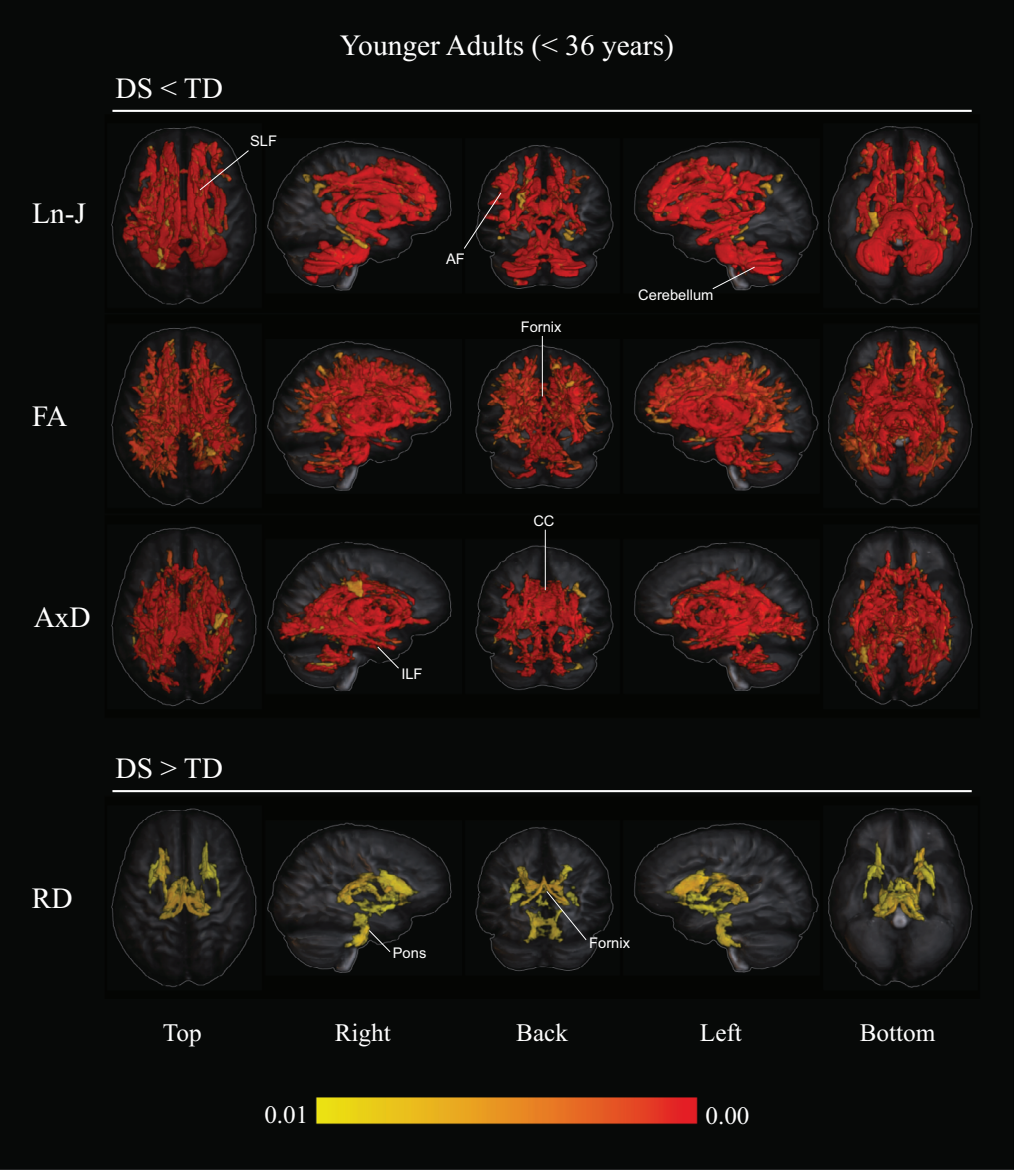
Overview of differences in Ln‐J and DTI metrics comparing younger adults with DS and younger TDs (*p* < 0.01). Voxel‐wise analysis revealed significant reductions in Ln‐J, FA, and AxD in younger DS compared to younger TDs (upper panels) and increases in RD in younger DS compared to younger TDs (lower panel). Age and sex were included as covariates. Results are visualized across multiple views (top, right, back, left, bottom), with a color bar indicating the statistical significance range (red = higher significance, lower *p* values). AF, arcuate fasciculus; AxD, axial diffusivity; CC, corpus callosum; DS, Down syndrome; DTI, diffusion tensor imaging; FA, fractional anisotropy; ILF, inferior longitudinal fasciculus; Ln‐J, determinant of Jacobian; RD, radial diffusivity; SLF, superior longitudinal fasciculus; TD, typically developing controls.

Table 2 summarizes all significant results (*p *< 0.01) observed in younger adults with DS compared to TDs. For a comprehensive comparison across metrics, Figure 1 visually encapsulates the observed differences in Ln‐J, FA, RD, and AxD. Detailed results for each diffusion metric are provided in Figures S1–S4 in supporting information.

**TABLE 2:** Between‐group differences in WM volume and DTI metrics (younger DS vs. younger TDs; *p* < 0.01).

Metric	Proportion of WM affected (%)	Affected areas in younger adults with DS
Ln‐J ↓	21.27	Anterior SLF I, right AF, body and anterior CIN, right temporal CIN, splenium, anterior limb of internal capsule, anterior ILF, pons, and cerebellar WM.
FA ↓	21.16	All major long association fibers, projection fibers, and cerebellar WM.
AxD ↓	13.65	CIN, genu and splenium of CC, internal capsule, ILF, posterior IFOF, a segment of UN, cerebral and cerebellar peduncles, and the cerebellar WM.
RD ↑	3.12	Anterior limb of internal capsule, right external capsule, fornix, and pons.

*Note*: Summary of significant WM volume and diffusion metric differences observed between younger adults with DS and typically developing controls. The “Proportion of WM Affected (%)” column indicates the percentage of voxels affected, calculated using the total WM mask as a reference.

Abbreviations: ↑, increase; ↓, reduction; AF, arcuate fasciculus; AxD, axial diffusivity; CC, corpus callosum; CIN, cingulate bundle; DS, Down syndrome; DTI, diffusion tensor imaging; FA, fractional anisotropy; IFOF, inferior fronto‐occipital fasciculus; ILF, inferior longitudinal fasciculus; Ln‐J, determinant of Jacobian; RD, radial diffusivity; SLF, superior longitudinal fasciculus; TD, typically developing controls; UN, uncinate fasciculus; WM, white matter.

### 3.3 Between‐group comparison results: older adults

In the older cohorts, whole‐brain analysis revealed significant Ln‐J reductions in the DS group compared to TDs (*p *< 0.01), spanning widespread brain areas in the frontal, temporal, and cerebellar regions. Significant FA reductions (*p *< 0.01) were also identified in the DS group, affecting all major long association fibers, projection fibers, the genu of the CC, and cerebellar WM.

Post hoc analyses showed increased MD values (*p *< 0.01) in the DS group compared to TDs, predominantly localized to the left frontoparietal connections, fornix, and projection tracts. Further analyses revealed significant RD increases (*p *< 0.01) in the DS group, affecting all major long association fibers, the fornix, projection fibers, and cerebellar WM. Additionally, AxD decreases (*p *< 0.01) were observed in the DS group, affecting temporal and deep WM regions.

**FIGURE 2:**
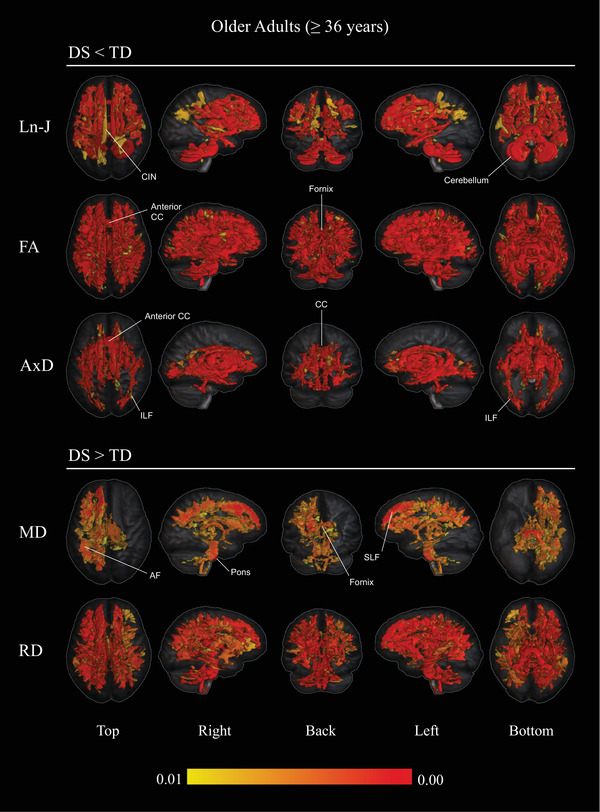
Overview of between‐group differences in Ln‐J and DTI metrics comparing older adults with DS and TD (*p* < 0.01). Voxel‐wise analysis revealed significant reductions in Ln‐J, FA, and AxD in DS compared to TD (upper panel) and increases in MD and RD in DS compared to TD (lower panel). Age and sex were included as covariates. Results are visualized across multiple views (top, right, back, left, bottom), with a color bar indicating the statistical significance range (red = higher significance, lower *p* values). AF, arcuate fasciculus; AxD, axial diffusivity; CC, corpus callosum; CIN, cingulum bundle; DS, Down syndrome; DTI, diffusion tensor imaging; FA, fractional anisotropy; ILF, inferior longitudinal fasciculus; Ln‐J, determinant of Jacobian; MD, mean diffusivity; RD, radial diffusivity; SLF, superior longitudinal fasciculus; TD, typically developing controls.

Table 3 summarizes all significant results (*p *< 0.01) observed in older adults with DS compared to TDs. Figure 2 provides a visual synthesis of all findings in the older DS and TD groups, including Ln‐J, FA, MD, RD, and AxD. Detailed results for each diffusion metric are presented in Figures S1–S5 in supporting information.

**TABLE 3:** Between‐group differences in WM volume and DTI metrics (older DS vs. older TDs; *p* < 0.01).

Metric	Proportion of WM affected (%)	Affected areas in older adults with DS
Ln‐J ↓	24.25	SLF I, II, AF, body and anterior CIN, genu, internal and capsule, anterior external capsule, a portion of the genu of CC, anterior ILF, cerebral peduncle, pons, cerebellar peduncle, and cerebellar WM.
FA ↓	35.40	All major long association fibers, projection fibers, fornix, genu of CC, and cerebellar WM.
MD ↑	12.36	Left SLF I and II, left AF, fornix, left anterior CIN, left temporal CIN, left anterior limb of internal capsule, left superior temporal gyrus, pons, and bilateral cerebral and inferior cerebellar peduncles.
RD ↑	26.12	All the frontoparietal connections with a left lateralization, body and temporal CIN, anterior limb of internal capsule, external capsule fornix, all main projection fibers, and cerebellar WM.
AxD ↓	12.92	CIN, genu, body, and splenium of CC, internal capsule, ILF, posterior IFOF, UN, cerebral and cerebellar peduncles, and the cerebellar WM.

*Note*: Summary of significant WM volume and diffusion metric differences observed between older adults with DS and typically developing controls. The “Proportion of WM Affected (%)” column indicates the percentage of voxels affected, calculated using the total WM mask as a reference.

Abbreviations: ↑, increase; ↓, reduction; AF, arcuate fasciculus; AxD, axial diffusivity; CC, corpus callosum; CIN, cingulate bundle; DS, Down syndrome; DTI, diffusion tensor imaging; FA, fractional anisotropy; IFOF, inferior fronto‐occipital fasciculus; ILF, inferior longitudinal fasciculus; Ln‐J, determinant of Jacobian; MD, mean diffusivity; RD, radial diffusivity; SLF, superior longitudinal fasciculus; TD, typically developing controls; UN, uncinate fasciculus; WM, white matter.

### 3.4 Within‐group comparison results

From the within‐group comparison of older and younger adults with DS, primary analyses revealed significant FA reductions (*p <* 0.01), particularly in frontoparietal connections, the arcuate fasciculus (AF), CC, fornix, and temporal WM in the older DS cohort. No significant differences in Ln‐J were observed at *p <* 0.01; however, trends at *p <* 0.05 were detected in the CC, projection fibers, and parietal WM, suggesting subtle volumetric differences that did not reach our predefined significance threshold.

Post hoc analyses identified increased RD (*p <* 0.01) in older adults with DS compared to younger adults with DS, largely overlapping with regions showing FA reductions, though with more pronounced left lateralization. While no significant differences in AxD or MD were found at *p <* 0.01, trends at *p <* 0.05 were observed in similar regions, suggesting potential age‐related changes that warrant further investigation.

For the within‐group comparison of older and younger TD adults, no significant differences (*p <* 0.01) were observed for any metric. However, FA reductions in the body of the CC and Ln‐J reductions within the thalamic nuclei were identified at *p <* 0.05. These findings, while notable, were excluded from the primary analysis, as only results at *p <* 0.01 were considered.

**FIGURE 3:**
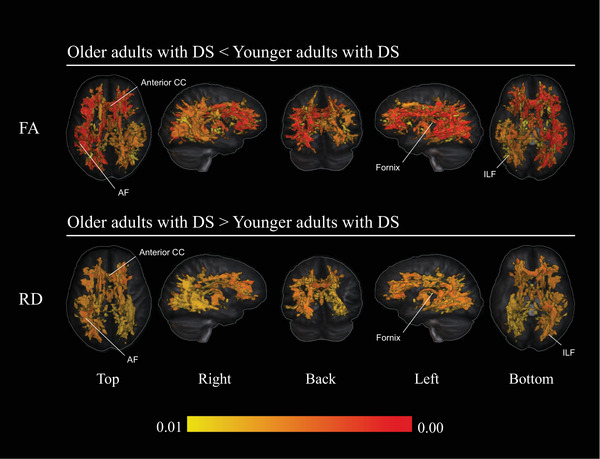
Overview of within‐group differences in DTI metrics comparing older adults with DS and younger adults with DS (*p *< 0.01). Voxel‐wise analysis revealed significant reductions in FA in older DS compared to younger DS (upper panel) and increases in RD in older DS compared to younger DS (lower panel). Sex was included as a covariate. Results are visualized across multiple views (top, right, back, left, bottom), with a color bar indicating the statistical significance range (red = higher significance, lower *p* values). AF, arcuate fasciculus; CC, corpus callosum; DS, Down syndrome; DTI, diffusion tensor imaging; FA, fractional anisotropy; ILF, inferior longitudinal fasciculus; RD, radial diffusivity

Table 4 summarizes all significant results (*p <* 0.01) from the within‐group comparisons in DS, while Figure 3 provides a comprehensive visual summary of these findings. Axial maps of FA and RD differences between older and younger adults with DS are presented in Figures S6 and S7 in supporting information.

**TABLE 4:** Within‐group differences in WM volume and DTI metrics (older DS vs. younger DS; *p* < 0.01).

Metric	Proportion of WM affected (%)	Affected areas in older adults with DS
FA ↓	14.18	SLF, body and genu of CC, left CIN, AF, fornix, ILF, and temporal WM.
RD ↑	11.13	SLF, body and genu of CC, left AF, fornix, ILF, and left temporal WM.

*Note*: Summary of significant WM diffusion metric differences observed between older and younger adults with DS from within‐groups analyses. The “Proportion of WM Affected (%)” column indicates the percentage of voxels affected, calculated using the total WM mask as a reference.

Abbreviations: ↑, increase; ↓, reduction; AF, arcuate fasciculus; CC, corpus callosum; CIN, cingulate bundle; DS, Down syndrome; FA, fractional anisotropy; ILF, inferior longitudinal fasciculus; RD, radial diffusivity; SLF, superior longitudinal fasciculus; WM, white matter.

## 4 DISCUSSION

In this study, we examined differences in WM volumetric and diffusion properties between younger and older adults with DS and TDs using D‐TBM analysis. Applied for the first time in adults with DS, this approach has been shown to significantly outperform TBSS in sensitivity and anatomical precision,^15^, ^16^ enabling whole‐brain and whole‐volume WM analysis, rather than being restricted to the FA skeleton. The analyses showed widespread anisotropy and WM volume reductions in DS participants compared to TDs from all ages, affecting major association fibers, the fornix, projection fibers, and cerebellar WM. These differences were evident across age groups, with additional within‐group comparisons highlighting further age‐related changes in DS. Aligning with previous dMRI studies (for a review, see^6^) the present findings suggest that the impact of an extra copy of chromosome 21 on neuronal connectivity is widespread and pervasive.

When older adults with DS were compared to sex‐ and age‐matched TDs, the proportion of WM showing reduced volume, low FA, and high RD was substantially greater than in younger adults with DS, while MD increases were observed exclusively in this age group. The volumetric reductions might reflect a neurodegenerative process, which may contribute to the increased isotropic diffusion and overall diffusivity in DS. Within‐group analyses also revealed widespread FA reduction and RD increase in the older DS group, affecting key regions such as frontal and temporal WM, areas linked to dementia symptoms in DS.^29^ While volumetric reductions were observed in key commissural fibers, projection fibers, and parietal WM at a more lenient statistical threshold (*p* < 0.05), these did not meet our predefined significance level of *p* < 0.01. This may reflect greater variability in volumetric measures compared to diffusion metrics, suggesting that microstructural alterations could be more consistently detectable than volume loss at this stage in DS.

In contrast, older TDs showed only a weak, non‐significant trend of localised FA changes, primarily in the CC, which did not meet our reporting threshold. These findings suggest that the extensive diffusion changes observed in DS are not merely reflective of typical aging but are likely indicative of AD neuropathology or accelerated aging processes, potentially indicating a substantial axonal degeneration in this population.

WM structures critical for memory and executive functions were particularly affected in DS. These included the frontoparietal and temporal long‐range association pathways, as well as the cingulum. While volumetric and FA changes in these areas were observed across both age groups, RD and MD increases were primarily evident in older DS, potentially suggesting more pronounced fiber degeneration with age.

While our study does not include direct AD biomarkers, the observed WM trajectory is consistent with the well‐established neuropathological progression of AD in DS. By the age of 40, nearly all individuals with DS exhibit hallmark AD pathology, including amyloid plaques and neurofibrillary tangles, even in the absence of clinical dementia.^2^, ^3^ Given this trajectory, the widespread WM degeneration seen in older adults with DS may reflect the impact of emerging AD pathology in this population.

Diffusion changes were also prominent in commissural structures, with distinct patterns in the anterior CC and the fornix. The anterior CC was affected only in older adults with DS and emerged as one of the main areas identified in within‐group analyses. These findings align with previous DS studies (for example^30^) and the known vulnerability of the CC in sAD,^31^, ^32^ highlighting it as a critical WM structure that may reflect shared neurodegenerative trajectories in DS‐related AD and sAD.

In contrast, the fornix was affected in both age groups and has rarely been reported in previous DS studies, likely due to its thin structure, which may go undetected by TBSS approaches. Therefore, its vulnerability may not be solely attributable to AD‐like degeneration but could reflect a more complex, DS‐specific pattern of WM changes.

Another key finding was the involvement of the left AF, a tract supporting language functions.^33^ While diffusion changes in this tract were evident even in younger individuals with DS, they became significantly more pronounced in the older cohort and were further confirmed by within‐group analyses. These findings suggest that WM changes in language‐critical tracts in DS may become increasingly prominent with age, potentially exacerbating language‐related impairments, already a core feature of the DS cognitive phenotype in the aging segment of this population.

### 4.1 Axial diffusivity reduction in DS

One of the most interesting results was the reduced AxD in individuals with DS compared to TDs across both age groups, particularly in the cingulum, CC, temporal, and cerebellar regions—areas critical for memory and language processes, which are especially relevant to the DS cognitive phenotype.^34^

AxD measures water diffusion along the principal tensor axis and is sensitive to axonal coherence and direction.^35^ This finding contrasts with previous studies, which reported increased AxD in this population^7^, ^36^ and with what would typically be expected in AD‐related neurodegeneration, in which WM loss would theoretically increase free water and thus AxD. Potential explanations for reduced AxD in DS may include early stages of WM inflammation or atypical WM development specific to this population.

Early neuroinflammatory processes, such as microglial activation and axonal swelling, have been shown to restrict water diffusion, particularly in the presence of reactive tissue changes.^37^, ^38^ This transient stage of inflammation may increase cellular density and introduce structural barriers, leading to reduced diffusivity before overt neurodegeneration occurs. Interestingly, reduced AxD has been reported in presymptomatic familial AD,^39^ mild cognitive impairment, and early AD,^40^ as well as in transgenic AD mouse models.^41^

Alternatively, reduced AxD in DS may reflect atypical WM development specific to this population. AxD differs across tissue types, increasing from GM to WM to CSF.^42^, ^43^ Consequently, reduced AxD in DS may indicate reduced WM organization or an increase in GM‐like partial volume within regions dominated by WM in typically developing individuals. Supporting this, WM volume in DS is consistently reduced compared to the general population and is disproportionately smaller than GM.^44^, ^45^ Additionally, within‐group comparisons of older and younger DS participants revealed no significant differences in AxD, suggesting that low AxD is relatively stable over time and less influenced by aging or AD progression.

In summary, low AxD in DS could arise either from early neuroinflammatory processes or a neurodevelopmental feature of trisomy 21. While our cross‐sectional design does not allow for definitive conclusions, these findings offer new insights into the microstructural profile of DS and highlight AxD as a potentially informative marker in this population. Future studies incorporating complementary modalities such as fluid‐attenuated inversion recovery imaging will be critical to disentangle neuroinflammatory and structural contributions to these alterations.

### 4.2 Increased RD and MD as markers of AD neuropathology in DS

The observed increases in MD and RD are likely closely associated with AD neuropathology in this population. MD changes were observed only in the older DS group, while RD increases were more widespread in older compared to younger DS participants. High RD was also observed when the older DS group was compared to the younger DS group. These metrics generally reflect changes in WM integrity, potentially linked to myelin degradation, axonal damage, and increased extracellular space.^35^, ^46^, ^47^, ^48^ Given the age range of the older DS cohort (36–58 years), these findings may potentially reflect AD‐related neurodegeneration. Accordingly, increases in RD and MD have been linked to Aβ deposition and cognitive impairment in the general population,^49^ and are common markers of neurodegeneration in sAD.^32^, ^50^, ^51^, ^52^

Therefore, increased RD and MD in older adults with DS may serve as potential markers of AD neuropathology, highlighting the progression of neurodegenerative changes in executive function and in language‐ and memory‐related WM networks—cognitive domains already vulnerable in those with DS and prone to AD‐related decline.

### 4.3 Conclusions

This study provides new insights into WM architecture in adults with DS using advanced registration techniques. Consistent with previous dMRI studies, we observed widespread FA reductions across age groups in DS, that may partially be explained by WM volume loss. In older adults with DS, the onset of AD neuropathology may have contributed to the increased extent of diffusion and volumetric changes in WM structures supporting memory, language, and executive functions. Notably, WM tracts vulnerable in sAD, such as the anterior CC, were significantly affected, suggesting shared neurodegenerative pathways between DS‐related AD and sAD.

This is the first study to report AxD reductions in DS adults, detected in temporal and callosal WM, potentially indicating early neuroinflammatory processes or atypical neurodevelopment in this population. In contrast, increases in RD and MD have been observed in older adults with DS and may suggest AD‐related neurodegeneration, particularly in frontoparietal, language, and memory networks critical for dementia in DS.

### 4.4 Limitations and future directions

This study used a full tensor‐based registration technique that outperforms traditional methods in anatomical accuracy, revealing previously unobserved patterns. However, one limitation is the voxel‐wise approach, which may not differentiate between widespread WM changes and tract‐specific alterations; integrating tractography could improve tract‐level precision. Additionally, while DTI provides high sensitivity, it is limited by partial volume effects and crossing fibers. Future studies could enhance specificity with multicompartmental models and fixel‐based or spherical deconvolution approaches to address these issues.

While we took extensive steps to minimize head motion‐related artifacts, we cannot completely rule out the possibility that motion may have had some influence on the results. However, head motion remains an inherent challenge in MRI studies of DS, and some degree of variability is unavoidable in this population.

The younger DS and TD groups differed in sample size; however, the older groups were well matched, and age and sex were carefully controlled across all analyses to minimize potential confounding effects. Last, the comparisons in older DS adults reflect both lifelong neurodevelopmental differences due to trisomy 21 and the effects of AD pathology. While this is an inherent challenge in DS research, future longitudinal studies may help disentangle these influences.

## CONFLICT OF INTEREST STATEMENT

The authors declare no conflicts of interest related to this work. Author disclosures are available in the supporting information.

## CONSENT STATEMENT

Informed consent was obtained from all participants or their legally authorized representatives prior to inclusion.

## Supporting information



Supporting Information

Supporting Information

## Data Availability

Our study reflects a commitment to diversity, equity, and inclusion. The research team, all based in London, comprises individuals of different nationalities, ethnicities, and career stages, ensuring varied perspectives. We included participants from a wide range of socio‐economic backgrounds and prioritized works from underrepresented voices in our references, ensuring equity throughout the research process.
