# Supplementary material for: White matter trajectories in Down syndrome and Alzheimer's disease: Insights from diffusion tensor–based morphometry
Source: Alzheimers Dement. 2025 Jun 12;21(6):e70382. doi: 10.1002/alz.70382 (PMC12162262; doi:10.1002/alz.70382)
Supplement: Supplementary file 1 — Supporting Information [file ALZ-21-e70382-s001.docx]

**Supplementary Figures and Legends**

**
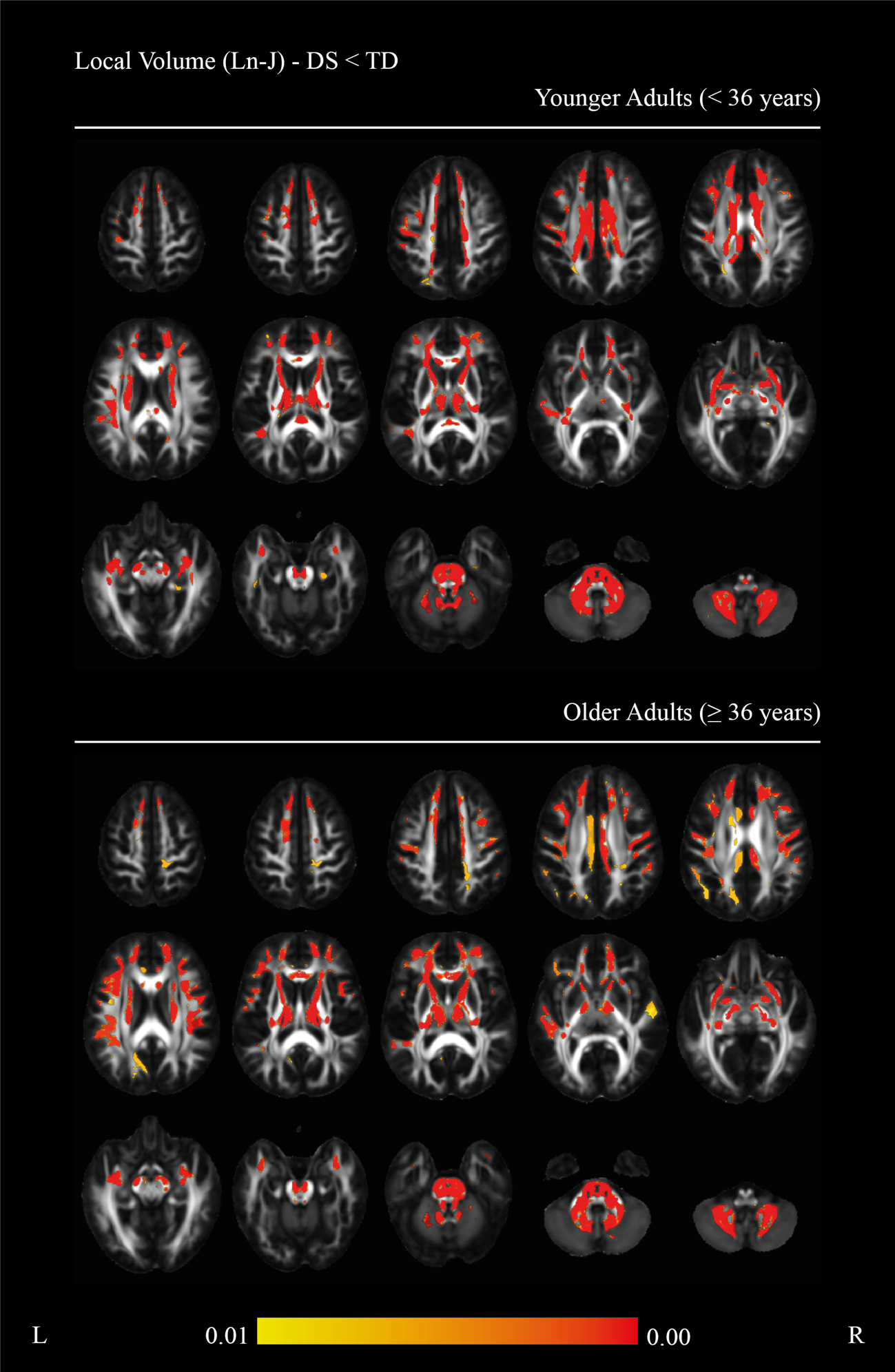
**

Supplementary Figure S1: Axial view of local volumetric reductions (Determinant of Jacobian; Ln-J) in Down syndrome (DS) compared to typically developing controls (TD) across both age groups (*P* < .01).

Whole-brain local volumetric reductions (ln-J) are visualised for younger adults with DS (<36 years) compared to TDs (upper panel) and older adults with DS (≥36 years) compared to TDs (lower panel). Significant differences (*P* < .01) are displayed on an axial slice map, with brighter colours indicating stronger differences (red = higher significance, lower p-values). L = Left, R = Right.

**
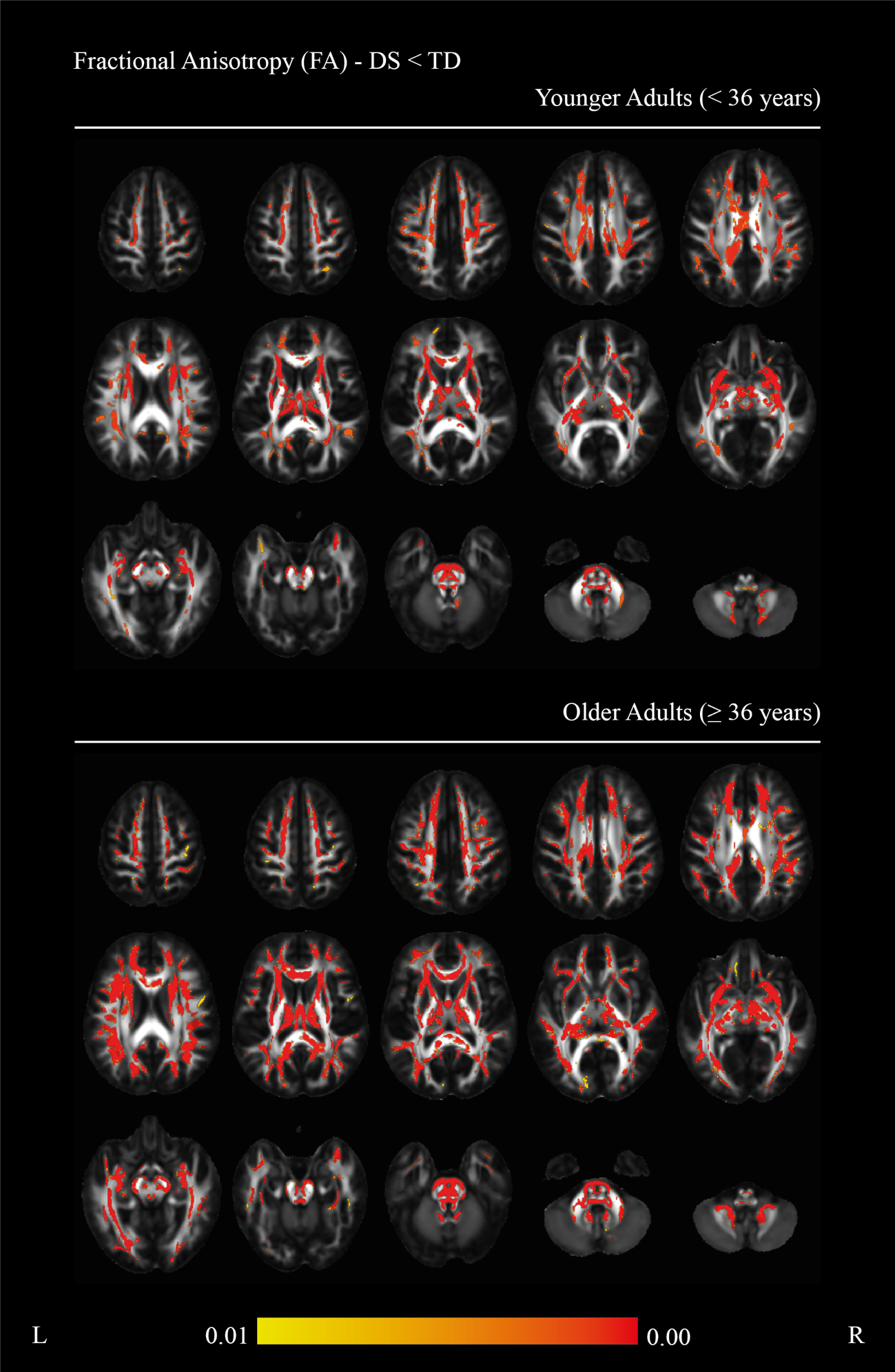
**

Supplementary Figure S2: Axial view of Fractional Anisotropy (FA) reductions in Down syndrome (DS) compared to typically developing controls (TD) across both age groups (*P* < .01).

Whole-brain reductions in white matter FA are visualised for younger adults with DS (<36 years) compared to TDs (upper panel) and older adults with DS (≥36 years) compared to TDs (lower panel). Significant differences (*P* < .01) are displayed on an axial slice map, with brighter colours indicating stronger differences (red = higher significance, lower p-values). The labels L and R denote the left and right hemispheres, respectively.

**
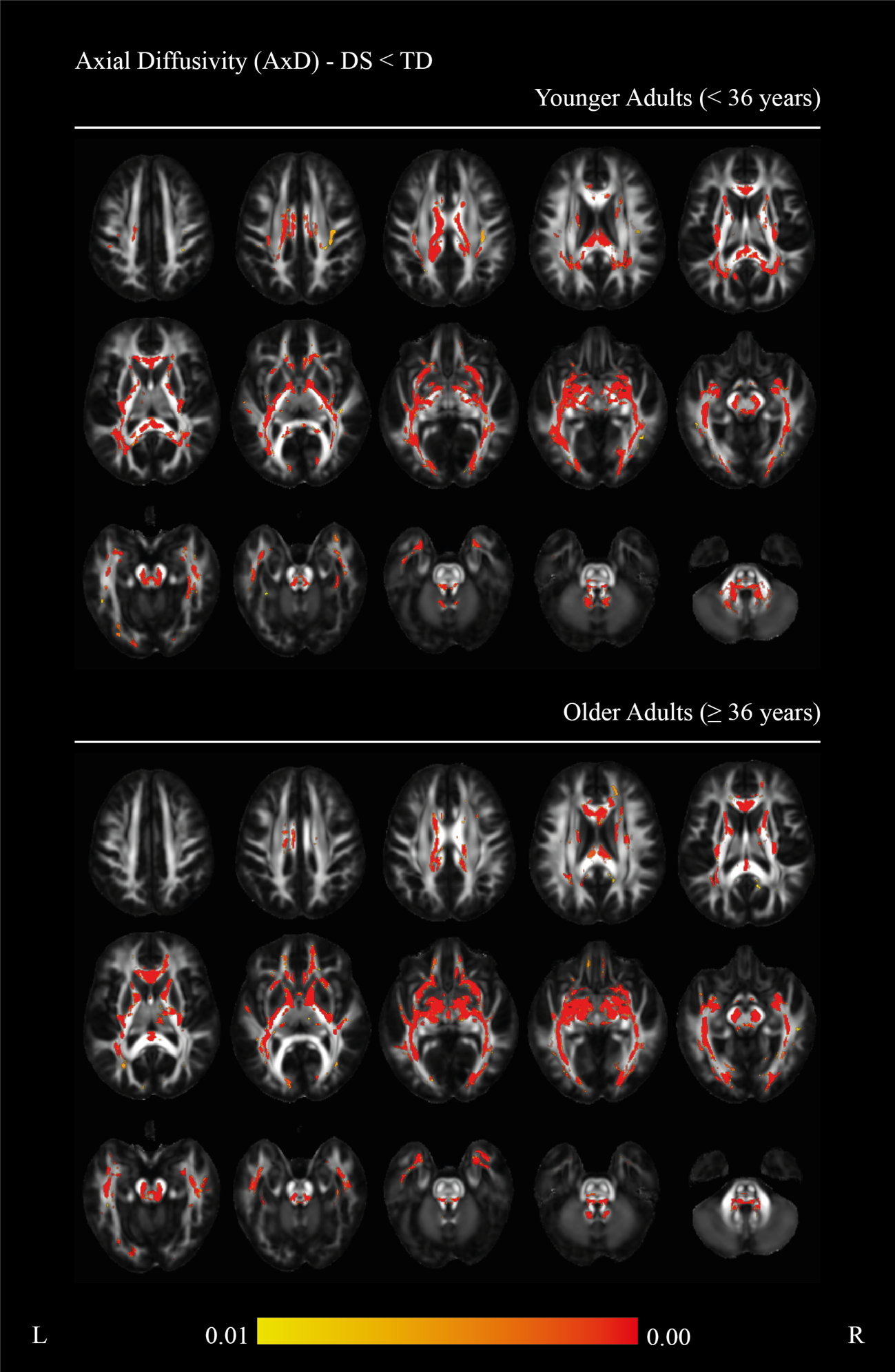
**

Supplementary Figure S3: Axial view of Axial diffusivity (AxD) reductions in Down syndrome (DS) compared to typically developing controls (TD) across both age groups (P < .01).

Whole-brain reductions in white matter AxD are visualised for younger adults with DS (<36 years) compared to TDs (upper panel) and older adults with DS (≥36 years) compared to TDs (lower panel). Significant differences (P < .01) are displayed on an axial slice map, with brighter colours indicating stronger differences (red = higher significance, lower p-values). The labels L and R denote the left and right hemispheres, respectively.

**
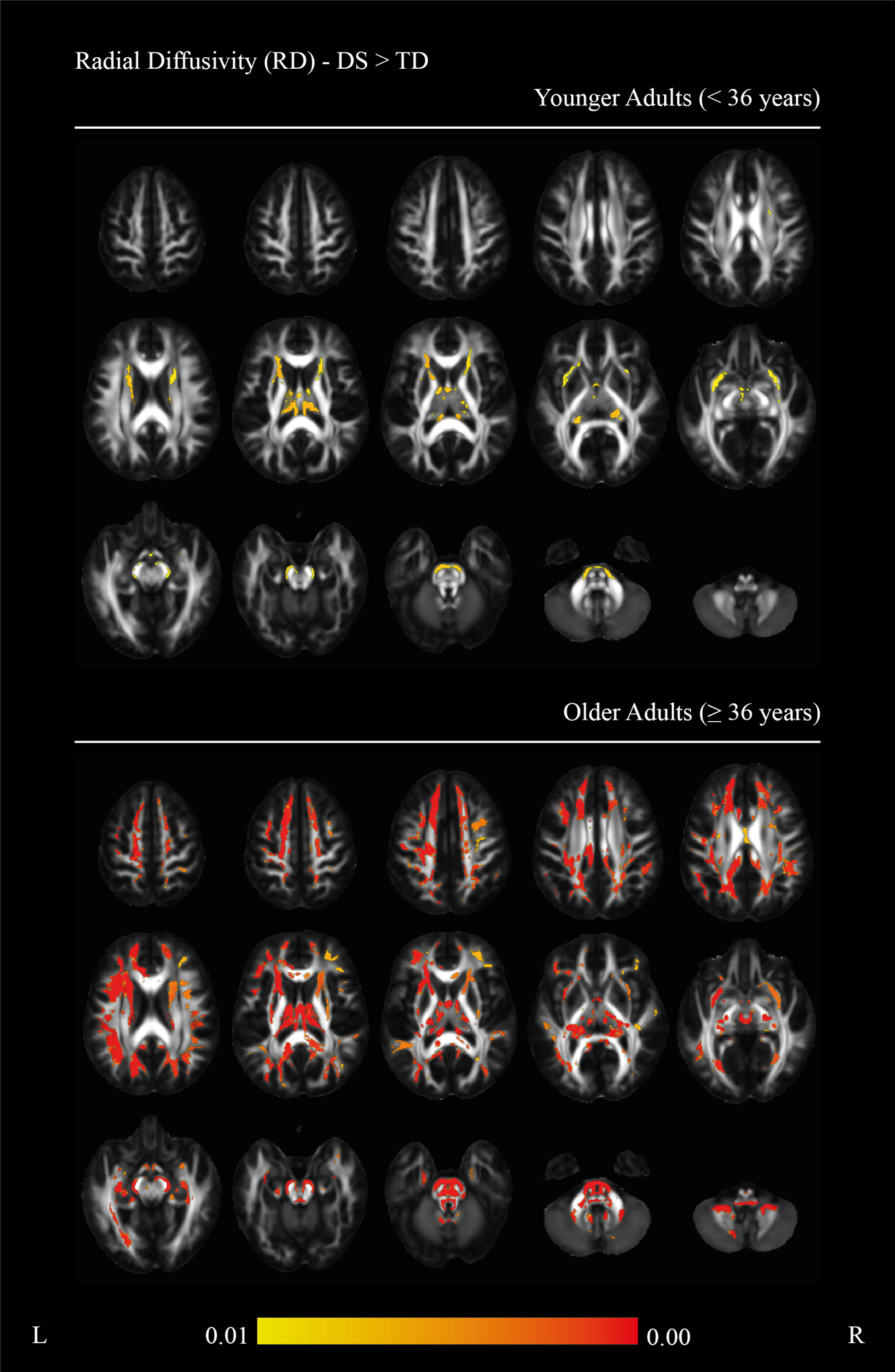
**

Supplementary Figure S4: Axial view of Radial diffusivity (RD) increases in Down syndrome (DS) compared to typically developing controls (TD) across both age groups (P < .01).

Whole-brain increases in white matter RD are visualised for younger adults with DS (<36 years) compared to TDs (upper panel) and older adults with DS (≥36 years) compared to TDs (lower panel). Significant differences (P < .01) are displayed on an axial slice map, with brighter colours indicating stronger differences (red = higher significance, lower p-values). The labels L and R denote the left and right hemispheres, respectively.

**
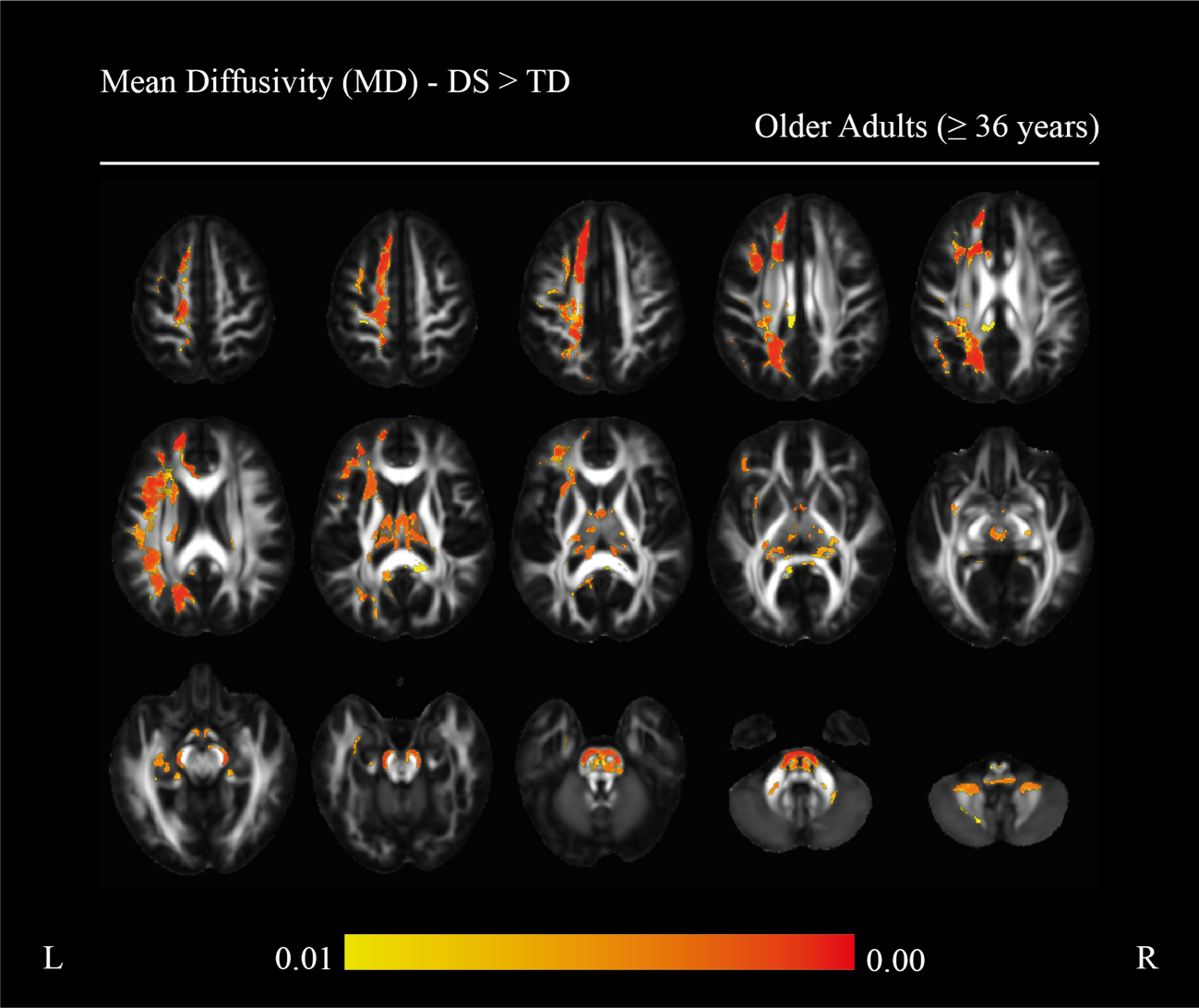
**

Supplementary Figure S5: Axial view of Mean diffusivity (MD) increases in older adults with Down syndrome (DS) compared to typically developing controls (TD) (P < .01).

Whole-brain increases in white matter MD are visualised for older adults with DS (≥36 years) compared to TDs. Significant differences (P < .01) are displayed on an axial slice map, with brighter colours indicating stronger differences (red = higher significance, lower p-values). The labels L and R denote the left and right hemispheres, respectively.

**
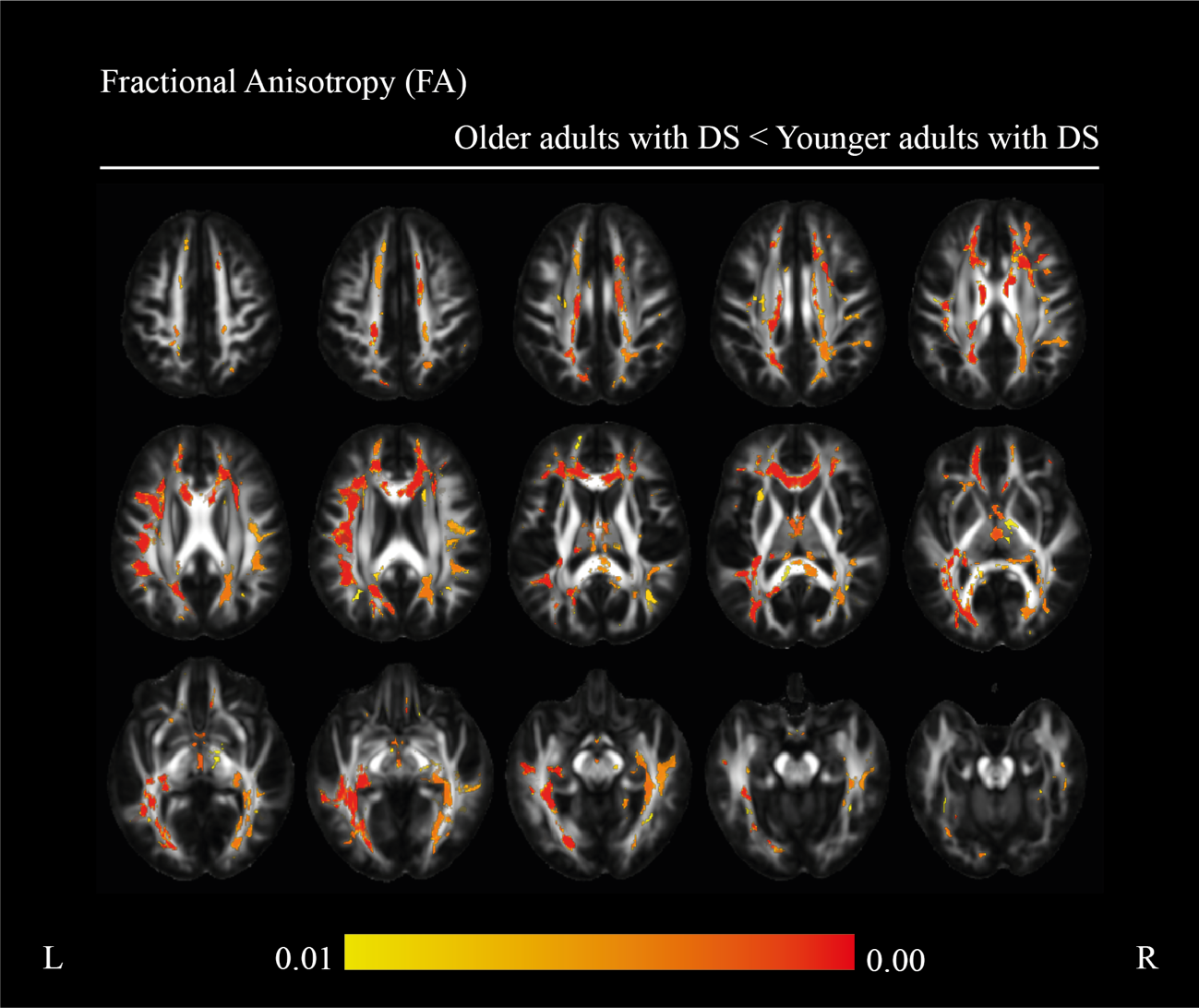
**

Supplementary Figure S6: Axial view of Fractional Anisotropy (FA) reductions in older adults with Down syndrome (DS) compared to younger adults with DS (P < .01).

Whole-brain reductions in white matter FA are visualised for older adults with DS (≥36 years) compared to younger adults with DS. Significant differences (P < .01) are displayed on an axial slice map, with brighter colours indicating stronger differences (red = higher significance, lower p-values). The labels L and R denote the left and right hemispheres, respectively.

**
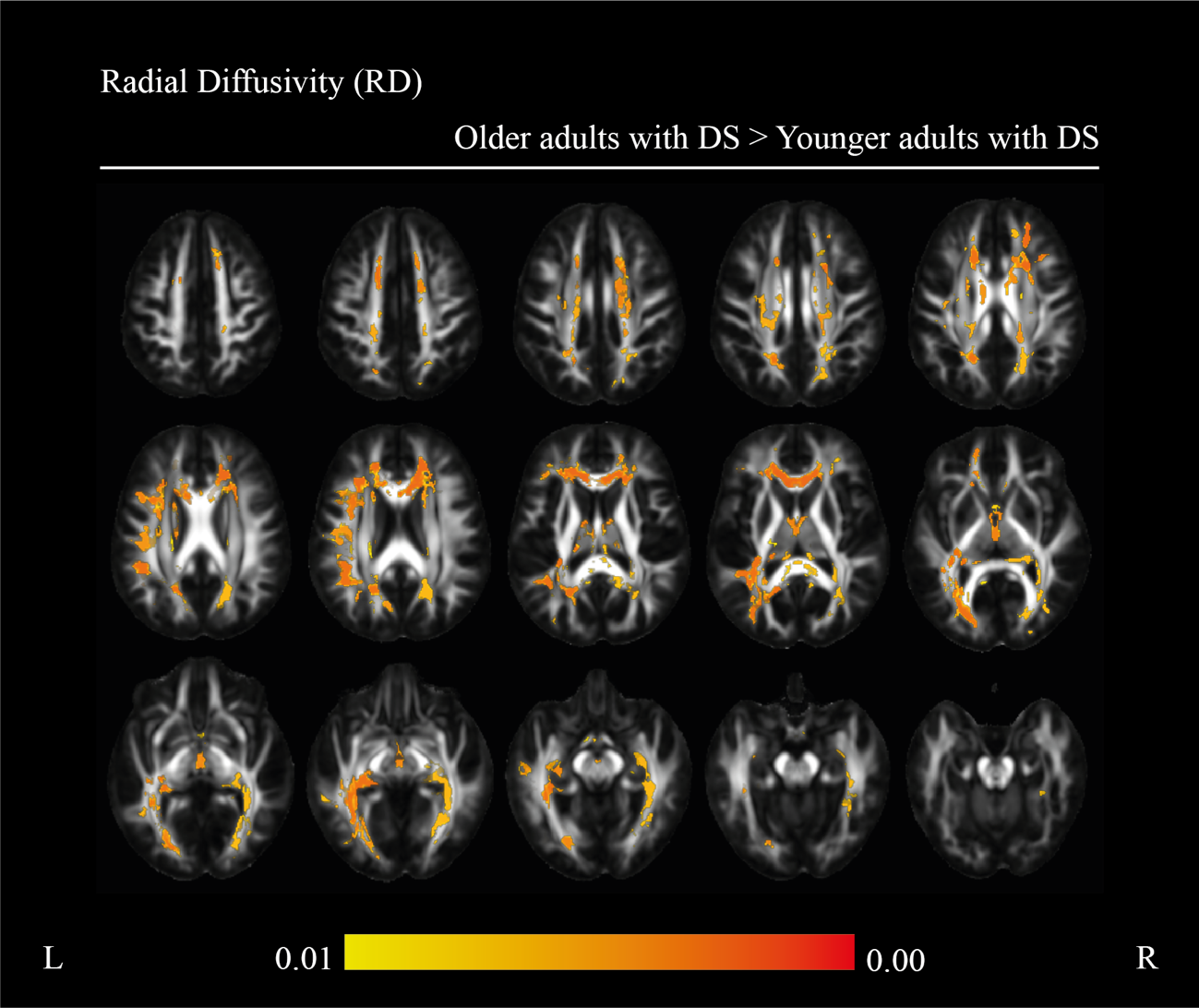
**

Supplementary Figure S7: Axial view of Radial diffusivity (RD) increases in older adults with Down syndrome (DS) compared to younger adults with DS (P < .01).

Whole-brain increases in white matter RD are visualised for older adults with DS (≥36 years) compared to younger adults with DS. Significant differences (P < .01) are displayed on an axial slice map, with brighter colours indicating stronger differences (red = higher significance, lower p-values). The labels L and R denote the left and right hemispheres, respectively.
